# Quantitation of endogenous GnRH by validated nano-HPLC-HRMS method: a pilot study on ewe plasma

**DOI:** 10.1007/s00216-022-04293-z

**Published:** 2022-09-05

**Authors:** Enrica Mecarelli, Riccardo Aigotti, Alberto Asteggiano, Paolo Giacobini, Manon Chasles, Yves Tillet, Federica Dal Bello, Claudio Medana

**Affiliations:** 1 Department of Molecular Biotechnology and Health Sciences, University of Turin, via Pietro Giuria 5, 10125 Turin, Italy; 2 University Lille, Inserm, CHU Lille, Laboratory of Development and Plasticity of the Neuroendocrine Brain, Lille Neuroscience & Cognition, Inserm UMR-S1172, 59000 Lille, France; 3 University of Tours, IFCE, Centre INRAE Val de Loire, 37380 Nouzilly, France

**Keywords:** GnRH, PNA, Nano-HPLC-HRMS, Method validation, Surrogate matrix, Endogenous peptide

## Abstract

Gonadotropin-releasing hormone isoform I (GnRH), a neuro-deca-peptide, plays a fundamental role in development and maintenance of the reproductive system in vertebrates. The anomalous release of GnRH is observed in reproductive disorder such as hypogonadotropic hypogonadism, polycystic ovary syndrome (PCOS), or following prenatal exposure to elevated androgen levels. Quantitation of GnRH plasma levels could help to diagnose and better understand these pathologies. Here, a validated nano-high-performance liquid chromatography–high-resolution mass spectrometry (HPLC-HRMS) method to quantify GnRH in ewe plasma samples is presented. Protein precipitation and solid-phase extraction (SPE) pre-treatment steps were required to purify and enrich GnRH and internal standard (*lamprey*-luteinizing hormone-releasing hormone-III, *l*-LHRH-III). For the validation process, a surrogate matrix approach was chosen following the International Council for Harmonisation (ICH) and FDA guidelines. Before the validation study, the validation model using the surrogate matrix was compared with those using a real matrix such as human plasma. All the tested parameters were analogous confirming the use of the surrogate matrix as a standard calibration medium. From the validation study, limit of detection (LOD) and limit of quantitation (LOQ) values of 0.008 and 0.024 ng/mL were obtained, respectively. Selectivity, accuracy, precision, recovery, and matrix effect were assessed with quality control samples in human plasma and all values were acceptable. Sixteen samples belonging to healthy and prenatal androgen (PNA) exposed ewes were collected and analyzed, and the GnRH levels ranged between 0.05 and 3.26 ng/mL. The nano-HPLC-HRMS developed here was successful in measuring GnRH, representing therefore a suitable technique to quantify GnRH in ewe plasma and to detect it in other matrices and species.

**Graphical abstract:**

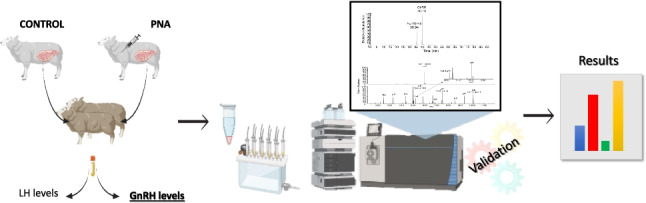

## Introduction

Gonadotropin-releasing hormone isoform I, hereafter GnRH, is a decapeptide playing a crucial role as a master regulator of reproductive functions in all vertebrate species studied so far [1, 2]. In mammals, GnRH-secreting neurons represent a small population of cells (few hundred/thousand) located in the hypothalamus, which release the decapeptide into the hypophyseal-portal circulation in a pulsatile manner [3, 4] and regulate the synthesis and secretion of luteinizing hormone (LH) and follicle-stimulating hormone (FSH) in the anterior pituitary, which finally modulate gonadal functions [5]. The GnRH secretion is pulsatile and its frequencies and amplitudes are shaped by circulating gonadal steroids (estradiol, progesterone, testosterone) and other neuroendocrine hormones [6, 7]. Alterations in GnRH secretion and/or GnRH signaling are associated with several reproductive dysfunctions in humans [8]. Circulating androgens during fetal life induce alteration of GnRH release in adult females as observed in women with PCOS (polycystic ovary syndrome) and in prenatally androgenized animal models [9]. Characterization of GnRH levels in different subjects with different biochemical conditions helps to understand both normal and pathophysiological states and it is useful for the diagnosis of complex diseases [10]. The difficulty in GnRH measurement in peripheral circulation relies on its short half-life, varying concentrations of GnRH depending on the estrous cycle stage for examples, and relatively low concentrations of the decapeptides. In the 1970s, several research groups carried out analytical determination to address this question and GnRH concentration was determined in human plasma based on age, sex, body mass, and clinical state using radioimmunoassay (RIA). These early experimental tests identified and quantified GnRH basal levels in reproductive age healthy women during follicular phase, children, and healthy men with values of 69.6, 30.0, and 67.9 pg/mL respectively [11]. Subsequent RIA studies have reported dissimilar values of GnRH plasma levels obtained from distinct type of subjects (i.e., men or women during menstrual cycle phases) with different extraction procedures [12]. For example, the maximum levels of GnRH detected in men peripheral blood samples were 9.5 pg/mL [13] and in plasma samples from women during mid cycle were 29 pg/mL [14] or 17 pg/mL [15]. However, GnRH levels were undetectable with the RIA assays in most of the studied subjects.

GnRH and analogous molecules (i.e., cetrorelix and ganirelix) are widely used as drugs in several conditions. Both agonists and antagonists of GnRH-related compounds are employed for pathologies such as PCOS and endometriosis, for assisted reproduction during infertility cases and as contraceptive methods [16]. Therefore, despite that RIA and immunoassays in general reach very low sensitivity with pg/mL limit of detection, they have a reduced specificity due to cross-reactivity [17], and consequently, analogues might represent a source of interference for immunochemical tests [18]. To our knowledge, no recent RIA methods were developed to quantify GnRH in biological samples. However, the immunoaffinity principle was employed in immunoaffinity capillary electrophoresis technique (IACE) and immunoaffinity chromatography (IAC) both coupled with mass spectrometry [19–21]. IACE and IAC have several drawbacks. IAEC suffers the unavailability of pre-assembled immunoaffinity devices with the antibody of interest. Consequently, the devices should be handmade by the analyst reducing the reproducibility of the analysis. For IAC, many immunoaffinity columns are commercially available but they are very expensive. Despite these disadvantages, both IAEC-MS and IAC-MS systems allow the detection and identification of several peptides including epitope sequences using different antibodies. In the last 20 years, efficient HPLC-(HR)MS methods have been developed to quantify proteins, peptides, and endogenous neuropeptides [22–28]. In order to achieve sensitive and selective quantitative data in targeted proteomic analysis, triple quadrupole in SRM mode or HRMS were typically used [29, 30]. Nowadays, a wide number of studies investigated peptides in biological samples; however, only few works [31–35] dealt with GnRH levels in biological matrices. Moreover, the use of HRMS allows to obtain higher sensitivity and specificity in discerning molecules with 500 k of resolving power and 3 ppm of mass accuracy [36].

Abundant protein depletion and quantitation of low-abundance proteins or peptides (LAPs, < 100 ng/mL) in biological fluids is a well-known challenge. To overcome this issue, several improvements in pre-treatment steps were carried out to enrich and simplify matrices using one or a combination of several clean-up approaches such as centrifuge filtration kits, solid-phase extraction, and immunoaffinity [37]. Another common issue of proteomics and in particular of biomarker discovery is the wide variability of procedures for sample handling and validation process [38]. Additionally, the best way to evaluate the accuracy and precision of a method for endogenous substances in biological matrices for clinical purposes is the use of certified reference materials (CRMs). Nevertheless, the development and evaluation of CRMs for peptide and protein analysis require an important endeavor making the process really troublesome. As an example, only recently, a new CRM for the well-known C-peptide was available [39]. Finally, a validation process is always required for the evaluation of the efficiency and reliability of the analytical developed methods.

The aim of this study was to present a validated nano-HPLC-HRMS method to quantify GnRH in biological fluids with special attention to ewe plasma. For the validation process, the surrogated matrix approach was selected as recommended by ICH and FDA guidelines [40, 41]. The surrogate matrix was here compared with a biological matrix such as human plasma to expand the application of the method also to pathological human plasma or biological fluid samples. The parameters of limits of detection (LOD) and quantitation (LOQ), lower LOQ (LLOQ) precision and accuracy, intra- and inter-day precision and accuracy, recovery, and matrix effect were assessed. The method was then applied for the quantitation of the neuropeptide in healthy and PNA ewe plasma samples. A preliminary goal of our study was to evaluate a possible correlation between measured GnRH and other circulating hormones, such as LH.

## Materials and methods

### Chemicals and reagents

Analytical standards (purity > 98%) of GnRH and *l*-LHRH-III were purchased from GeneCust (Chalmont, France). Stock solutions were prepared with a concentration of 1000 ng/mL using methanol and were stored at − 20 °C until use. Further dilutions were obtained in 0.1% formic acid in water using plastic vials in order to avoid adsorption. All aqueous solutions were prepared with HPLC-grade water from a Milli-QAcademic System (Millipore, Burlington, MA, USA). Acetonitrile hyper-grade for LC–MS, formic acid, and TFA (trifluoroacetic acid) were purchased from VWR International (Radnor, PA, USA); acetic acid and SigMatrix Serum Diluent were purchased from Merck (Darmstadt, Germany). Human plasma samples were provided by “Centro Produzione e Validazione Emocomponenti (C.P.V.E.) SC Banca del Sangue, Città della Salute e della Scienza” Torino, Italy. The fresh human plasma refrigerated was maintained at the temperature of + 4 °C until use. We employed ten different lots of human plasma refrigerated, previously analyzed to detect traces of GnRH. Each lot was used for the validation protocol only in the case of undetectable GnRH (< LLOQ, vedi infra).

### Animal and sample collection

#### Animals

PNA (prenatal androgen) ewes (*n* = 8) were generated by injecting pregnant mothers twice a week from the 65th day of gestation to the 105th day of gestation with 100 mg of testosterone propionate diluted in sesame oil. The control group (*n* = 8) received sesame oil only according to previously validated protocols [42, 43]. Ewes were born between the 7^th^ and the 17^th^ of September 2018; within 24 h after birth, animals were separated from their mother and fed artificially with free access to artificial milk. After, weaning females were fed daily with barley straw, lucerne hay, and commercial concentrate, with free access to water and mineral blocks.

Experiments were conducted on Ile de France ewes provided by the INRAE experimental unit UEPAO Val de Loire (Indre et Loire, France—latitude 47° 32 N and longitude 0° 46E, https://doi.org/10.15454/1.5573896321728955E12). All procedures were performed in compliance with the European directive 2010/63/EU on the protection of animals used for scientific purposes and were approved by the local ethical committee for animal experimentation (CEEA VdL, Tours, France, n 2,016,062,917,335,667).

#### Blood sampling

Blood samples were taken through a jugular catheter from PNA and control ewes. Blood samples in heparinized tubes were centrifuged at 3000 rpm for 15 min and the plasma was stored (not more than 1 month) at − 20 °C until LH and GnRH assays.

#### LH assay

LH concentrations were determined by using double-antibody ELISA immunoassays developed in our laboratory [44]. The sensitivity of the assay was 0.1 ng/mL.

### Sample extraction and purification

For GnRH sample extraction and purification, we adapted the protocol described in a previous work by Tata and co-workers [45] for the cetrorelix (a GnRH synthetic analogous) quantitation in dams brain and fetal brain samples. Briefly, 250 µL of ewe plasma and human plasma or surrogate matrix (SigMatrix Serum Diluent) for calibration curves was treated, after fortification with 30 ng/mL of IS, with 400 µL of formic acid in cold acetonitrile and incubated for 15 min at − 20 °C for protein precipitation step. After centrifugation (20 min, 4 °C, 15,000 g), the supernatant was freeze-dried using a CentriVap (Labconco Co., Kansas City, MO, USA) and reconstituted with 1 mL of 3% acetic acid and 1% TFA water solution, hereafter loading solution (LS). The reconstituted sample was sonicated and centrifuged for 15 and 10 min, respectively. The supernatant was purified with a solid-phase extraction (Strata-X 33 µm Polymeric Reversed Phase, 60 mg/3 mL, Phenomenex, Bologna, Italy). The cartridges were equilibrated with 3 mL of methanol and 3 mL of LS and, after loading samples, washed with 3 mL of LS/methanol (70:30 v/v), and eluted with 3 mL of methanol and 3% acetic acid (70:30 v/v). The eluted solution was freeze-dried using a CentriVap and reconstituted with 100 µL 0.1% formic acid in water ultra-pure solution and injected into the nano-HPLC-HRMS system. All samples were prepared and analyzed in duplicate.

### Nano-HPLC-HRMS setting

Separation and analysis were achieved using a nano-HPLC-HRMS instrument. The nano-HPLC system consisted of a Thermo Dionex Ultimate 3000 chromatograph coupled for identification and quantitation to an Orbitrap Fusion analyzer (Thermo Scientific, Milan, Italy).

The chromatographic separation was achieved with a PepMap™ RSLC C18 column (2 µm, 100 Å, 75 µm × 50 cm; Thermo Scientific, Milan, Italy) preceded by a nano-pre-concentration column (C18 PepMap trap cartridge 100 Å, 5 µm, 0.3 mm × 5 mm; Thermo Scientific, Milan, Italy). Eluents were 0.1% formic acid in water (A) and 0.1% formic acid in acetonitrile/water 8/2 (B) for the separation column, and TFA 0.05% in water/acetonitrile 98/2 (P) for the pre-concentration one. The run gradient started from 5% of B maintained for 8 min (to pre-concentrate), and increased to 45% of B in 45 min up to 90 in 2 min. This final washing step was maintained for 13 min. Then, the column went back to the initial conditions in 2 min and reconditioned for 30 min. The flow was set to 300 nL/min; the injection volume was 3 µL. Nano-column has a low capacity and with complex matrices, such as plasma and other biological fluids; it is not possible to inject more than 3–5 µL to avoid saturation of active sites of the stationary phase. The pre-concentration step was reached with 100% of P at a flow rate of 5 µL/min and lasted 8 min. It was conducted in a backflush mode and the pre-concentration column went back to the initial condition 20 min before the end of the separation run.

The PepMap column was connected to a nano-ESI source set with the following parameters: spray positive voltage 2000 V and ion transfer tube temperature 275 °C. Full-scan spectra were acquired in the range of *m/z* 300–2000 with a resolution of 60 k. MS^2^ spectra of analyte (*m/z* 591.8, *z* = 2) and IS (*m/z* 630.3, *z* = 2) protonated molecular ions were acquired in the range of *m/z* 100–1300 with a resolution of 30 k. CID activation mode was used, with quadrupole as isolation mode and 1.6 Da as isolation window. CID collision energy was set at 28% and it was optimized with a direct injection experiment of analytical standards of GnRH and LHRH-III. We used the MS^2^ dedicated experiments performed in CID activation mode to quantify GnRH and *l*-LHRH-III in all the matrices. The mass accuracy of recorded ions (vs. calculated) was ± 2 milli mass units (without internal calibration).

## Method validation

A full single-laboratory and single-operator validation process was performed following guidelines proposed by the ICH [40] and FDA [41]. The validation parameters LOD, LOQ, LLOQ, linearity, intra- and inter-day accuracy and precision, selectivity, recovery, matrix effect, and carry-over were evaluated. For method validation, we used two different matrices: surrogate matrix and human plasma. The first was used as a calibrant matrix and the calibration curve was prepared fortifying it with GnRH at the following concentrations: 0.08–0.1–0.5–1-5–10-30 ng/mL with IS at final concentration 30 ng/mL. Human plasma was used both as real matrix to evaluate the surrogate matrix reliability and as quality control matrix. Quality control (QC) samples was prepared using human plasma with no detectable GnRH amount fortified at low (LQC), medium (MQC), and high (HQC) concentration levels (0.1–1-10 ng/mL). Hereafter, the human plasma samples have to be intended as human plasma with undetectable GnRH amount (< LLOQ), except when precisely annotated. Other QC samples for the evaluation of recovery were prepared using control ewe plasma with undetectable amount of GnRH spiked with a low concentration level (0.1 ng/mL).

LOD and LOQ were calculated as three and ten times, respectively, the calculated standard deviation of signal in zero samples (sample fortified with only IS). We evaluated and compared LOD and LOQ measurements obtained in the surrogate matrix (SM) and human plasma (HP). For LLOQ measurement, we also evaluated the accuracy and precision.

Linearity of calibration curves was evaluated using XLStat® (version 23.4.1203.0, 2021) software, and the best-weighted linear regression model was applied. The intra (repeatability)- and inter (reproducibility)-day precision and accuracy were estimated in QC samples at three concentration levels (L/M/HQC) plus zero-QC and were repeated 5 times in 3 days.

The ICH recommends evaluating selectivity for endogenous analytes analyzing the samples with the use of a discriminative detection system. The parameter, usually acceptable when lower than 25% of the LLOQ signal, was here tested using a high-resolution mass spectrometer (Orbitrap Fusion) with MS/MS experiments. Both analyte signals in the surrogate matrix and human plasma were assessed.

Recovery and carry-over were estimated with a post-extraction fortification of QC samples at three concentration levels of GnRH (L/M/HQC) and analyzing solvent (0.1% formic acid in water ultra-pure solution) after the analysis of a high concentration level of GnRH (30 ng/mL), respectively.

For matrix effect, we considered the comparison between the signals of GnRH obtained in solvent vs. QC samples both spiked with different concentrations of GnRH (L/M/HQC).

Finally, we also evaluated the chromatography efficiency analyzing the precision (RSD%) of the retention time of GnRH and IS in one (*n* = 10) and 3 runs (*n* = 50) for intra- and inter-run precision respectively.

All the analyses for validation purposes were done three times.

## Results

### HRMS characterization of the studied molecules

**Fig. 1:**
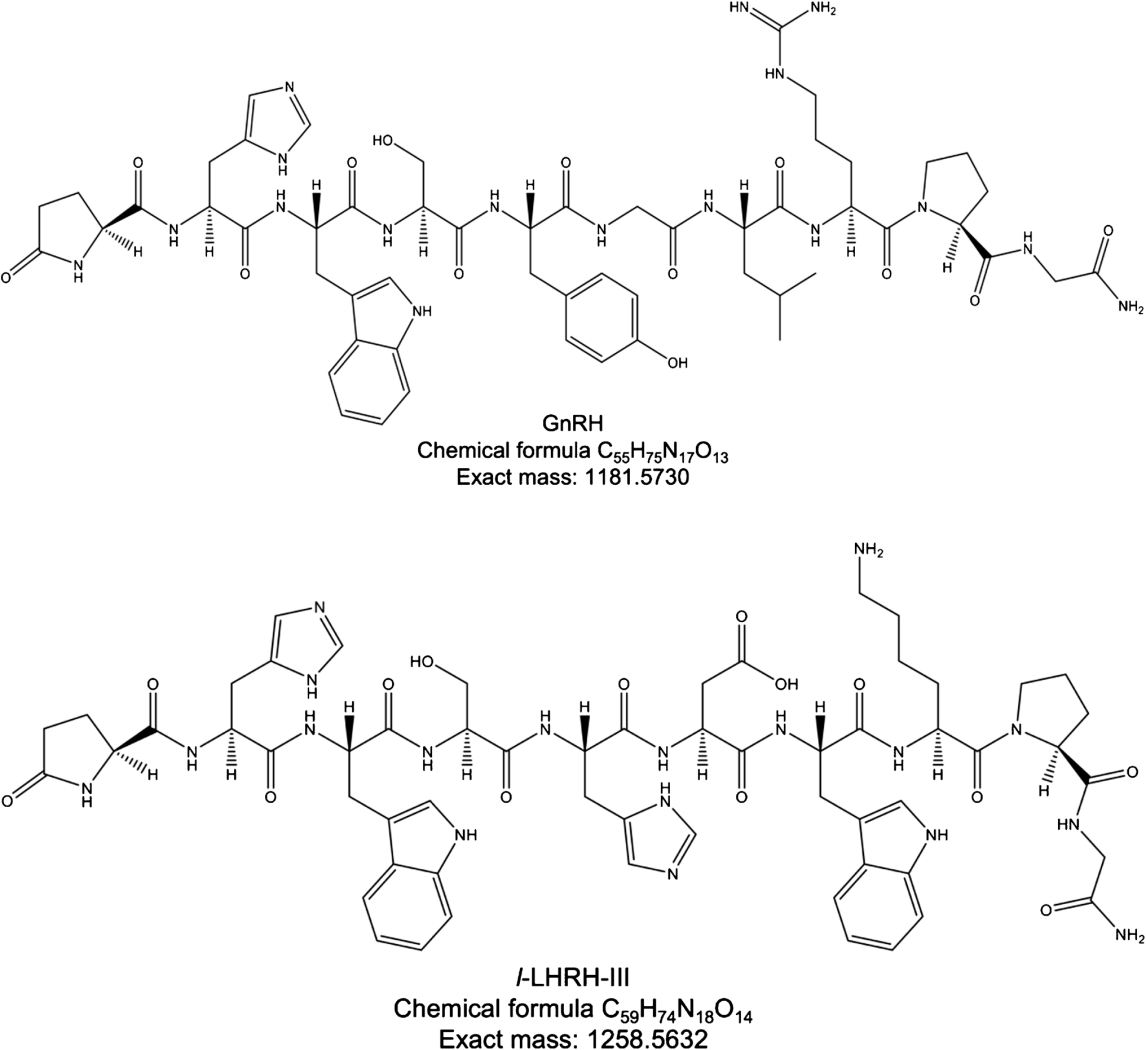
Structure of GnRH (top) and its lamprey variant *l*-LHRH-III (bottom)

**Fig. 2:**
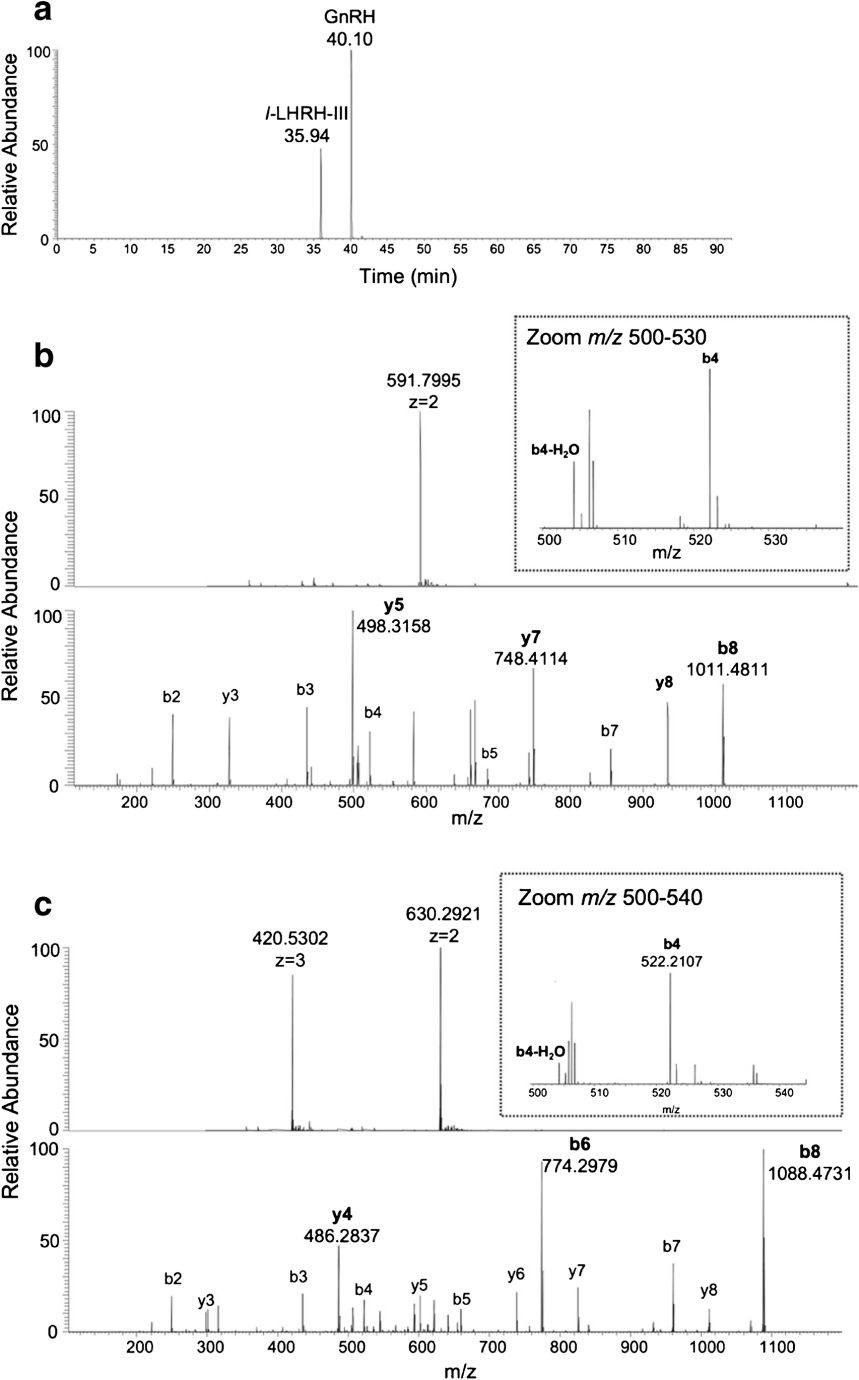
Chromatographic separation (**a**), full-mass spectra with bi- and tri-charged precursor and MS/MS spectra of GnRH, 10 ng/mL (**b**) and *l*-LHRH-III, 30 ng/mL (**c**). The zoomed boxes in Figure 2b and 2c show the ion b4-H_2_O (*m/z*=504.21990) for GnRH and *l*-LHRH-III useful to identify analogue molecules

For the quantitation of GnRH and *l*-LHRH-III (Fig. 1), we previously characterized their mass spectra acquired in high-resolution mode (*R* = 50 K). The molecules eluted (Fig. 2a) at 35.94 min (*l*-LHRH-III) and 40.10 min (GnRH). As shown at the bottom of Fig. 2b, c, after the ionization process, the full-scan spectra present a double-charged ion at *m/z* 591.7938, *z* = 2 for GnRH, and bi- and tri-charged ions at *m/z* 630.2889, *z* = 2 and 420.5302, *z* = 3 for *l*-LHRH-III. The double-charged species for both analytes were selected as precursor ions of MS^2^.

Tandem mass spectrum acquired in CID activation mode for GnRH showed as most intense product ions the y5 (*m/z* = 498.3147), y7 (*m/z* = 748.4100), and b8 (*m/z* = 1011.4795) fragments. Other abundant product ions (with *z* = 1) were as follows: y3 (*m/z* = 328.2092), b3 (*m/z* = 435.1775), b4 (*m/z* = 522.2096), b5 (*m/z* = 685.2729), b7 (*m/z* = 855.3784), and y8 (*m/z* = 934.4894).

For *l*-LHRH-III, the most abundant product ions as Fig. 2 shows were b8 (*m/z* = 1088.4767) and b6 (*m/z* = 774.3004). As for GnRH, many other product ions were present in MS^2^ spectra: b2 (*m/z* = 249.0982), y3 (*m/z* = 300.2030), b3 (*m/z* = 435.1775), y4 (*m/z* = 486.2860), b4 (*m/z* = 522.2096), y5 (*m/z* = 601.3093), b5 (*m/z* = 659.2685), y6 (*m/z* = 738.3682), y7 (*m/z* = 825.4002), b7 (*m/z* = 960.3747), and y8 (*m/z* = 1011.4697). As noticed by other work, the isoforms of GnRH of different shared the product ions b4 (*m/z* = 522.2096) and b4-H_2_O (*m/z* = 504.21990) useful to identify analogue molecules [31]. The sum of y5, y7, and b8 product ion AUCs (areas under the curves) was used to quantify GnRH in samples.

### Validation of the method

The nano-HPLC-HRMS method validation was performed with a surrogate matrix for calibration curves and with human plasma for quality control analysis. The results of the validation process are shown in Table 1.

**Table 1:** Validation parameters for GnRH

Parameters	Validation results	
LOD (ng/mL)	0.008 (SM)	
	0.009 (HP)	
LOQ (ng/mL)	0.024 (SM)	
	0.028 (HP)	
LLOQ 0.08 ng/mL	BIAS%	RSD%
	22.5	17.5
Intra-day (*n* = 3)		
	BIAS%	RSD%
0.1 ng/mL	16.3	26.3
1 ng/mL	20.4	21.6
10 ng/mL	19.0	19.0
Inter-day (*n* = 15)		
	BIAS%	RSD%
0.1 ng/mL	− 13.1	11.0
1 ng/mL	− 18.0	25.1
10 ng/mL	21.0	12.7
Selectivity	17.3% (SM)	
	23.8% (HP)	
Recovery (*n* = 3)		
0.1 ng/mL	63.1%	
1 ng/mL	80.1%	
10 ng/mL	77.0%	
Matrix effect (*n* = 3)		
0.1 ng/mL	77.5%	
1 ng/mL	58.2%	
10 ng/mL	74.4%	

LOD and LOQ values for GnRH reached in the surrogate matrix were 0.008 and 0.024 ng/mL, respectively. In the human plasma, the concentrations for LOD and LOQ were 0.009 and 0.028 ng/mL. The lower limit of quantitation (LLOQ) was also evaluated for the surrogate matrix and it corresponded to the low concentration point of the calibration curve (0.08 ng/mL reduced to 0.032 ng/mL following the sample preparation) with accuracy and precision of 22.5% and 17.5%, respectively.

The linearity of the calibration curve was assessed using a regression plot and the curves resulted heteroscedastic. Then, we investigated 1/*x*, 1/*y*, 1/*x*^2^, and 1/*x*^0.5^ as weight coefficients of the regression model using XLStat® software, and 1/*x*^0.5^ was the best weight coefficient giving minimal standard relative error value and an *R*-squared value > 0.99.

The accuracy and precision of intra-day analysis estimated at three concentration levels of GnRH in the QC samples (L/M/HQC) ranged between 16.3 and 20.4%, and 19.0 and 26.3% respectively. The same was applied for the inter-day tests and the results, depicted in Table 1, ranged between 18.0 and 21.0% for accuracy, and 11.0 and 25.1% for precision.

The selectivity in the not-spiked surrogate matrix and human plasma at the retention time of the analyte were 17.3% and 23.8% of the analyte response at the LLOQ level, respectively.

The obtained recovery values in QC samples were 63.1%, 80.1%, and 77.0% for low, medium, and high levels respectively. We checked the recovery also in three real QC samples obtained from control ewe plasma, fortified with 0.1 ng/mL, and the results were similar to the one obtained in the surrogate matrix (65.4%). No carry-over was observed analyzing a blank solution after the higher level of the calibration curve.

Matrix effect percentage assessed at three concentration levels of GnRH (L/M/HQC) was 77.5%, 58.2%, and 74.4%, respectively.

Finally, the intra-run precision of retention time of GnRH and IS was 0.47% and 0.46%, respectively, and the inter-run precision of GnRH retention time was 1.59% and 1.18% for IS.

### Application of the method to ewe plasma samples and results

The validated method was applied to real biological samples belonging to a group of healthy and PNA ewes. The quantitation of GnRH was achieved in all the analyzed samples and the results, as the average values of duplicate analysis, are shown in Table 2 together with LH assay measurements (“LH assay”).

**Table 2:** Ewe plasma analysis results of GnRH and LH

Sample name	GnRH level (ng/mL)	LH level (ng/mL)	Sample name	GnRH level (ng/mL)	LH level (ng/mL)
Ewe_Control_1	0.09	0.10	Ewe_PNA_1	1.17	0.10
Ewe_Control_2	0.22	0.30	Ewe_PNA_2	0.05	0.10
Ewe_Control_3	0.09	0.30	Ewe_PNA_3	0.12	0.40
Ewe_Control_4	0.47	0.10	Ewe_PNA_4	0.23	0.10
Ewe_Control_5	0.21	0.10	Ewe_PNA_5	3.16	5.00
Ewe_Control_6	0.15	1.10	Ewe_PNA_6	0.41	0.60
Ewe_Control_7	0.14	0.10	Ewe_PNA_7	0.17	0.40
Ewe_Control_8	0.17	0.10	Ewe_PNA_8	3.26	0.40
Average	0.19	0.28	Average	1.07	0.89

**Fig. 3:**
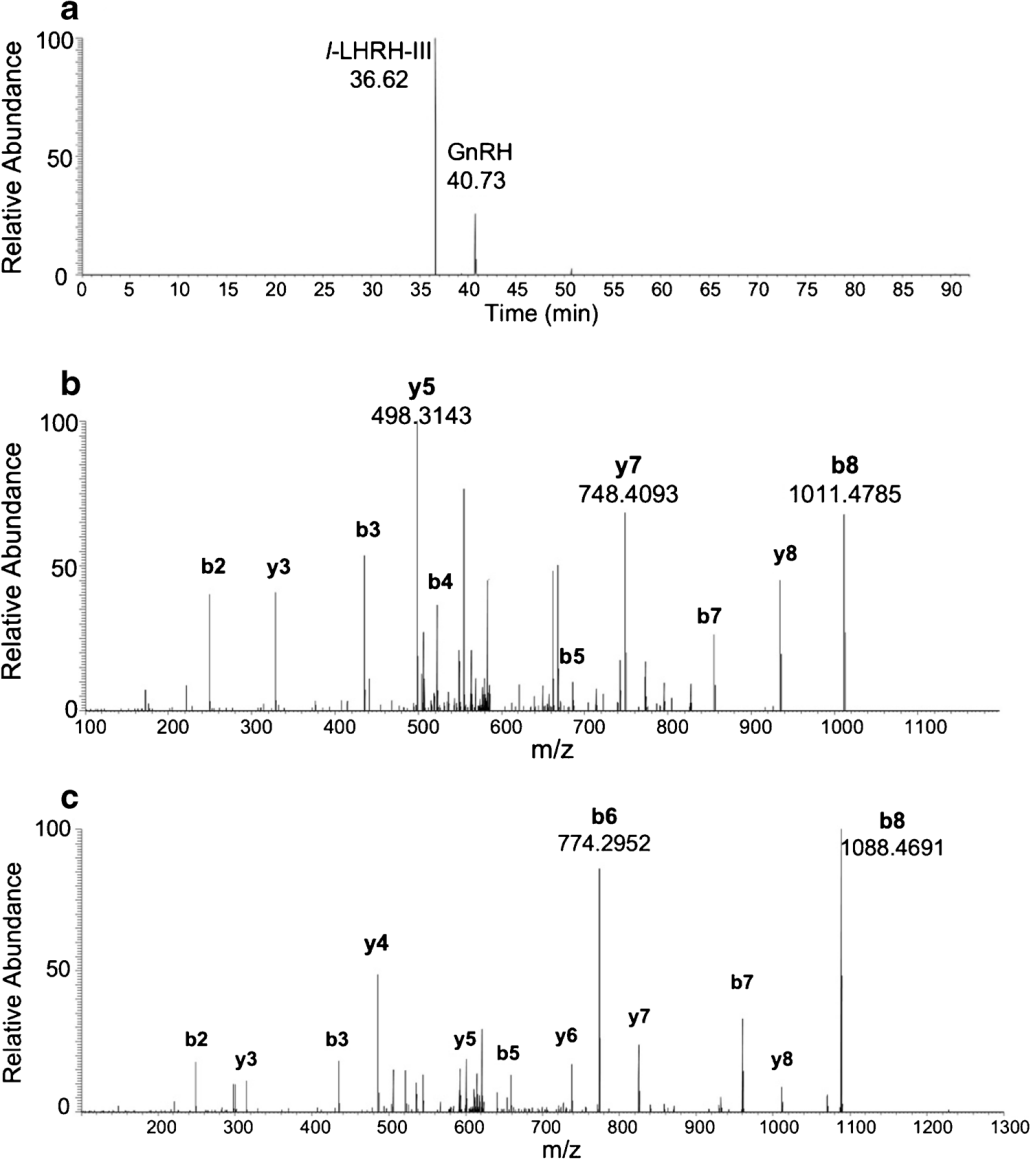
Chromatographic separation (**a**), MS/MS spectra of GnRH, 3.26 ng/mL (**b**), and *l*-LHRH-III, 30 ng/mL (**c**) in ewe PNA sample number 8 (Ewe_PNA_8)

The control (*n* = 8) group presented an average concentration of GnRH and LH of 0.19 and 0.28 ng/mL respectively; in contrast, the PNA (*n* = 8) group showed an average concentration of GnRH and LH of 1.07 and 0.89 ng/mL, correspondingly. In Fig. 3, the GnRH and IS chromatograms and MS/MS spectra with fragmentation patterns in the real pathological sample are shown.

## Discussion

### Method validation discussion

For qualitative and quantitative analyses of endogenous compounds, such as GnRH, ICH guidelines propose four different approaches: (i) standard addition; (ii) background subtraction; (iii) surrogate matrix; and (vi) surrogate analytes. We selected the third approach, the surrogate matrix, because the first one is a very time-consuming method; the surrogate analyte for GnRH is difficult and expensive to obtain and the background substation strictly depends on the biological matrix used [46]. Moreover, we selected the surrogate artificial matrix, excluding the 0.1% formic acid water solution, the stripped matrix, and the plasma belonging to other non-mammalian species because these are not sufficient compared with the analyzed sample matrices, that is, ewe plasma. The chosen surrogate matrix of choice might mimic the real biological one, demonstrating the similitude of the two by the evaluation of the linearity of the calibration curves, the recovery, and the matrix effect of the sample preparation. In our study, we compared the surrogate matrix with human plasma used as a real biological matrix. As previously described, the lots of human plasma used for the comparison had an undetectable level of GnRH (< LLOQ, 0.08 ng/mL). Human plasma was used as the substrate for the standard addition approach fortified with GnRH at the same concentration used in the case of the calibration curve (IS 30 ng/mL). The linearity of calibration curves was obtained in the two matrices evaluating the slopes of the weighted linear regression (with equation *y* = *mx* + *q*). The slope values were *m*_surrogate matrix_ = 0.226 and *m*_human plasma_ = 0.356, respectively. Thus, the concentration of endogenous GnRH in human plasma samples was assessed using the standard addition approach and the calculated concentration value (0.469 ng/mL) was compared with that calculated using the surrogate matrix calibration curve obtaining a mean value of 0.433 ng/mL. The difference between the two results was 7.7%. The two methodologies gave a comparable calculated concentration of endogenous GnRH confirming the eligibility of the surrogate matrix to quantify the neuropeptide. Finally, recovery and matrix effect values measured at the medium concentration QC level (1 ng/mL) were 70.0% and 58.7% for surrogate matrix, and 80.1% and 58.2% for human plasma, and also in this case, the values were comparable. Hence, the parameters of linearity, recovery, and matrix effect in the two matrices matched.

With the developed and validated method, we obtained suitable values of LOD (0.008 SM ng/mL) and LOQ (0.024 SM ng/mL) to quantify GnRH in biological samples of ewe plasma. There are no international recommended criteria for these parameters and their values should be able to identify the lower acceptable limits of measurements above the background signal. The LLOQ corresponded to the lower concentration level of the calibration curve, 0.08 ng/mL. With respect to the works of Wang et al. [32] and Bussy et al. [34], the limits of detection and quantitation here obtained with the use of high-resolution mass spectrometry and injecting 3 µL of samples were comparable. Hence, the efficiency and reliability of the nano-HPLC-HRMS analytical method here presented were satisfactory. Other research groups achieved a LOD of approximately 1 to 5 pg/mL, quantifying GnRH as equine doping drug after an external administration [35, 47].

For method validation, we followed FDA and ICH protocols as previously described. The guidelines indicate a ± 15% limit for accuracy and precision of inter-/intra-day analysis for small molecules, but other research articles on peptides and proteins reported an acceptance level of ± 30% [48–50]. Our obtained results were acceptable because of below 30%. The maximum deviation was achieved for intra-day LQC level RSD (26.3%). It is important to highlight that we analyzed 4 concentration levels (zero/L/M/HQC) and the intra-day was to intend as intra-day of sample preparation, since the nano-HPLC separation run and the analysis set last 92 and 1840 (approx. 31 h) min, respectively.

Since we used the MS^2^-dedicated experiments to quantify the analyte in the QC samples and in the surrogate matrix, the achieved selectivity was satisfactory (17.3% in SM and 23.8% in QC) and no interferences were found in both matrices. This approach was the same used in other proteomics/peptidomics studies where the analytes were quantified with an ion trap MS/MS experiment or with a triple quadrupole mass analyzer in MRM mode [51–53].

Recovery and matrix effect results were acceptable and consistent with an average of 73.4% and 70.0% respectively. ICH does not mention limits for recovery and matrix effect but, at all the concentration levels, the extent of recovery of the analyte should be constant, and the matrix effect should be controlled. Although the sample preparation and purification steps were quite long (3 days) and challenging (many solvent exchanges, SPE, etc.), the validated method demonstrated to have a good recovery, but most importantly, that it was repeatable, as suggested also by other studies [54, 55]. No carry-over was observed using the surrogate matrix and QCs, indicating that during the nano-HPLC-HRMS analysis no further blank samples were required. By analyzing the results of the nano-chromatographic separation, satisfactory reproducibility and robustness were shown. Despite some other studies stating that the nano-HPLC system is not reproducible and less satisfactory compared with HPLC methods [56–58], we obtained very good values for intra- and inter-day precision of chromatographic retention time. These results were obtained because in the room where the instrument was maintained at a controlled environmental temperature (20 ± 2 °C), both the nano-column and pre-concentration column were thermostated (45 °C) and we used the pick-up injection mode for injecting 3 µL of samples.

### Biological sample result discussion

As previously explained, the quantitation of GnRH in biological samples was obtained by weighting the area values of the calibration curve in the surrogate matrix for 1/*x*^0.5^. It is worthy of note that in all of the analyzed samples the GnRH was detected, in some cases at a very low concentration (i.e., 0.05 ng/mL in PNA ewes 2). As regards plasma storing, we did not perform the long-term stability or heparinized sample stability of GnRH. However, the pioneering work of Alain Caraty [59] regarding the collection and measurement of GnRH levels in the serial portal blood of ram was performed on animals treated with a very high level of heparin: 25,000 UI first and 5000 UI each 30 min for 7–8 h. The serial sampling of portal blood consisted in collecting blood by draining a hemorrhage after a physical lesion of the portal blood vessels. From this protocol, we supposed that heparin should not affect the GnRH stability.

The first evidence was that the average amount of the neuropeptide in the ewe control group was quite lower compared to the PNA group (0.19 vs. 1.07 ng/mL). The amounts of LH in the studied ewes indicated the same trend (control 0.28 vs. PNA 0.89 ng/mL). Some other studies in the literature suggested a correlation with a prenatal androgen exposure and the changing in hormonal levels [9, 60]. Future determination of the variation of hormone concentration during the ovulatory cycle might provide the kinetics of hormonal release correlating GnRH and LH secretion.

The results of PNA group samples showed a very high variation, from 0.05 to 3.26 ng/mL: this evidence may be related to the characteristic pulsatile release of GnRH [3, 4]; the GnRH level will vary if the samples occur during or between pulses’ variability. It could be also related to the phenotype of the ewes. The same phenomenon was observed in the control group, although with a lower variation range (from 0.09 to 0.47 ng/mL).

## Conclusion

In conclusion, a nano-HPLC-HRMS for the quantitation of GnRH in biological matrices was developed and validated with good results. The method was selective, sensitive, and robust and it was successfully applied to real biological samples.

Following the ICH and FDA guidelines, a full single-laboratory and single-operator validation process was completed evaluating the fundamental parameters of validation. The surrogate matrix approach selected for the validation protocol gave comparable results regarding the slope of the calibration curve, the recovery, and the matrix effect related to a real biological matrix, such as human plasma with no detectable GnRH levels. The endogenous concentrations of GnRH in human plasma samples, if present, were quantified using the surrogate or human plasma matrix and they were comparable (0.469 vs. 0.433 ng/mL).

The LOD (0.008 ng/mL) and LOQ (0.024 ng/mL) values achieved were suitable to allow the quantitation of neuropeptides in biological matrices. Certainly, a very sensitive analytical method to measure the endogenous levels of peptides (or other biomolecules) is mandatory due to their low abundance.

The achieved accuracy and precision of the intra- and inter-day analyses guaranteed good method reliability with satisfactory repeatability and reproducibility of the results.

The recovery percentage obtained with sample purification and enrichment pre-analytical steps here presented, albeit these were quite long and difficult with many analytical operations, was satisfactory and allowed both the extraction and the quantitation of GnRH in analyzed samples. The application of the analytical method here presented and validated to ewe plasma samples confirmed its ability to quantify a very low amount of the neuropeptide in the biological matrix and pave the way for the application of this method to the quantitation of GnRH in human samples. GnRH could become an eligible candidate as a biomarker for the early diagnosis of reproductive distress in the human population.

## Data Availability

The data reported in this manuscript that support the findings of this study are available on request from the corresponding author on reasonable request.

## References

[1] Millar RP, Kastin AJ (2013). GnRH (LHRH). Handbook of biologically active peptides.

[2] White BA, Portefield SP, White BA, Portefield SP (2013). Hypothalamus-pituaitary complex. Endocrine and reproductive physiology.

[3] Levine JE, Pau KYF, Ramirez VD, Jackson GL (1982). Simultaneous measurement of luteinizing hormone-releasing hormone and luteinizing hormone release in unanesthetized, ovariectomized sheep. Endocrinology.

[4] Caraty A, Orgeur P, Thiery JC (1982). Demonstration of the pulsatile secretion of LH-RH into hypophysial portal blood of ewes using an original technic for multiple samples. C R Seances Acad Sci III.

[5] Knobil E (1990). The GnRH pulse generator. Am J Obstet Gynecol.

[6] Clarke IJ (2011). Control of GnRH secretion: one step back. Front Neuroendocrinol.

[7] Marshall JC, Dalkin AC, Haisenleder DJ, Paul SJ, Ortolano GA, Kelch RP (1991). Gonadotropin-releasing hormone pulses: regulators of gonadotropin synthesis and ovulatory cycles. Recent Prog Horm Res.

[8] Sanchez-Garrido MA, Tena-Sempere M (2020). Metabolic dysfunction in polycystic ovary syndrome: pathogenic role of androgen excess and potential therapeutic strategies. Mol Metab.

[9] Yan X, Yuan C, Zhao N, Cui Y, Liu J (2014). Prenatal androgen excess enhances stimulation of the GNRH pulse in pubertal female rats. J Endocrinol.

[10] Lewandowski KC, Cajdler-Łuba A, Ireneusz S, Bieńkiewicz M, Lewiński A (2011). The utility of the gonadotrophin releasing hormone (GnRH) test in the diagnosis of polycystic ovary syndrome (PCOS). Polish Journel Endocrinol.

[11] Keye WR, Kelch RP, Niswender GD, Jaffe RB (1973). Quantitation of endogenous and exogenous gonadotropin releasing hormone by radio-immuno assay. J Clin Endocrhwl Metab.

[12] Teuwissen B, Fauconnier JP, Thomas K (1978). Radioimmunoassay of LH RH. Application to human plasma. Gynecol Obstet Invest.

[13] Jeffcoate SL, Fraser MH, Holland DT, Gunn A (1974). Radio-immuno assay of luteinizing-releasing hormone (LH-RH) in serum from man, sheep and rat. Acta Endocrinol (Copenh).

[14] Saito S, Musa K, Oshima I, Yamamoto S, Funato T (1975). Radioimmunoassay for luteinizing hormone releasing hormone in plasma. Endocrinol Jpn.

[15] Arimura A, Kastin AJ, Schally AV (1974). Immunoreactive LH-releasing hormone in plasma: midcycle elevation in women. Clin Endocrinol Metab.

[16] Kumar P, Sharma A (2014). Gonadotropin-releasing hormone analogs: understanding advantages and limitations. J Hum Reprod Sci.

[17] Tate J, Ward G (2004). Interferences in immunoassay. Clin Biochem Rev.

[18] Doding S (2009). Interferences in quantitative immunochemical methods. Medica, Biochem.

[19] Guzman NA (2001). Determination of immunoreactive gonadotropin-releasing hormone in serum and urine by on-line immunoaffinity capillary electrophoresis coupled to mass spectrometry. J Chromatogr B Biomed Sci Appl.

[20] Guzman NA, Blanc T, Phillips TM (2008). Immunoaffinity capillary electrophoresis as a powerful strategy for the quantification of low-abundance biomarkers, drugs, and metabolites in biological matrices. Electrophoresis.

[21] Hoos JS, Sudergat H, Hoelck JP, Stahl M, De Vlieger JSB, Niessen WMA, Lingeman H, Irth H (2006). Selective quantitative bioanalysis of proteins in biological fluids by on-line immunoaffinity chromatography-protein digestion-liquid chromatography-mass spectrometry. J Chromatogr B Anal Technol Biomed Life Sci.

[22] van den Broek I, Sparidans RW, Schellens JHM, Beijnen JH (2008). Liquid chromatography/tandem mass spectrometric method for the quantification of eight proteolytic fragments of ITIH4 with biomarker potential in human plasma and serum. Rapid Commun Mass Spectrom.

[23] Bredeho M, Schanzer W, Thevis M (2008). Quantification of human insulin-like growth factor-1 and qualitative detection of its analogues in plasma using liquid chromatography/electrospray ionisation tandem mass spectrometry. Rapid Commun Mass Spectrom.

[24] Galli S, Naranjo A, Van Ryn C, Tilan JU, Trinh E, Yang C, Tsuei J, Hong SH, Wang H, Izycka-Swieszewska E, Lee YC, Rodriguez OC, Albanese C, Kitlinska J (2016). Neuropeptide Y as a biomarker and therapeutic target for neuroblastoma. Am J Pathol.

[25] Grangeon A, Clermont V, Barama A, Gaudette F, Turgeon J, Michaud V (2019). Development and validation of an absolute protein assay for the simultaneous quantification of fourteen CYP450s in human microsomes by HPLC-MS/MS-based targeted proteomics. J Pharm Biomed Anal.

[26] Wang J, Ge M, Sun L, Ahmed I, Li W, Lin H, Lin H, Li Z (2021). Quantification of crustacean tropomyosin in foods using high-performance liquid chromatography–tandem mass spectrometry method. J Sci Food Agric.

[27] Kim MK, Lee TH, Suh JH, Eom HY, Min JW, Yeom H, Kim U, Jung HJ, Cha KH, Choi YS, Youm JR, Han SB (2010). Development and validation of a liquid chromatography-tandem mass spectrometry method for the determination of goserelin in rabbit plasma. J Chromatogr B Anal Technol Biomed Life Sci.

[28] van den Broek I, Sparidans RW, Schellens JHM, Beijnen JH (2008). Quantitative bioanalysis of peptides by liquid chromatography coupled to (tandem) mass spectrometry. J Chromatogr B Anal Technol Biomed Life Sci.

[29] Stahl-zeng J, Lange V, Ossola R, Eckhardt K, Krek W, Aebersold R, Domon B (2007). High sensitivity detection of plasma proteins by multiple reaction monitoring of N -glycosites. Mol Cell Proteomics.

[30] Thomas A, Kohler M, Schänzer W, Delahaut P, Thevis M (2011). Determination of IGF-1 and IGF-2, their degradation products and synthetic analogues in urine by LC-MS/MS. Analyst.

[31] Myers TR, Gabor P (2006). A new strategy utilizing electrospray ionization-quadrupole ion trap mass spectrometry for the qualitative determination of GnRH peptides. J mass Spectrom.

[32] Wang H, Chung-Davidson YW, Li W (2014). Identification and quantification of sea lamprey gonadotropin-releasing hormones by electrospray ionization tandem mass spectrometry. J Chromatogr A.

[33] Yao JF, Zhou N, Bai L, Xu PX, Liu KL, Xue M (2014). Simultaneous determination of five novel luteinizing hormone-releasing hormone antagonists by LC-MS and pharmacokinetics in rats following cassette dosing. J Chromatogr B Anal Technol Biomed Life Sci.

[34] Bussy U, Wang H, Chung-Davidson YW, Li W (2015). Simultaneous determination of gonadotropin-inhibitory and gonadotropin-releasing hormones using ultra-high performance liquid chromatography electrospray ionization tandem mass spectrometry. Anal Bioanal Chem.

[35] Richards SL, Cawley AT, Cavicchioli R, Suann CJ, Pickford R, Raftery MJ (2016). Aptamer based peptide enrichment for quantitative analysis of gonadotropin-releasing hormone by LC-MS/MS. Talanta.

[36] Senko MW, Remes PM, Canterbury JD, Mathur R, Song Q, Eliuk SM, Mullen C, Earley L, Hardman M, Blethrow JD, Bui H, Specht A, Lange O, Denisov E, Makarov A, Horning S, Zabrouskov V (2013). Novel parallelized quadrupole/linear ion trap/orbitrap tribrid mass spectrometer improving proteome coverage and peptide identification rates. Anal Chem.

[37] Millioni R, Tolin S, Puricelli L, Sbrignadello S, Fadini GP, Tessari P, Arrigoni G (2011) High abundance proteins depletion vs low abundance proteins enrichment: Comparison of methods to reduce the plasma proteome complexity. PLoS One 6: . 10.1371/journal.pone.0019603 10.1371/journal.pone.0019603 PMC3087803 21573190

[38] Chandramouli K, Qian P-Y (2009) Proteomics: challenges, techniques and possibilities to overcome biological sample complexity. Hum Genomics Proteomics 1: . 10.4061/2009/239204 10.4061/2009/239204 PMC2950283 20948568

[39] Moore M, Dougall T, Ferguson J, Rigsby P, Burns C (2017). Preparation, calibration and evaluation of the First International Standard for human C-peptide. Clin Chem Lab Med.

[40] International Council for Harmonisation of Technical Requirements for Pharmaceuticals for Human Use (2019) Bioanal Method Valid. M10

[41] U.S. Department of Health and Human Services F and DA (2018) Bioanalytical Method Validation Guidance for Industry. US Dep Heal Hum Serv Food Drug Adm. 1–41

[42] Robinson JE, Forsdike RA, Taylor JA (1999). In utero exposure of female lambs to testosterone reduces the sensitivity of the gonadotropin-releasing hormone neuronal network to inhibition by progesterone. Endocrinology.

[43] Monniaux D, Genêt C, Maillard V, Jarrier P, Adriaensen H, Hennequet-Antier C, Lainé AL, Laclie C, Papillier P, Plisson-Petit F, Estienne A, Cognié J, di Clemente N, Dalbies-Tran R, Fabre S (2020). Prenatal programming by testosterone of follicular theca cell functions in ovary. Cell Mol Life Sci.

[44] Faure MO, Nicol L, Fabre S, Fontaine J, Mohoric N, McNeilly A, Taragnat C (2005). BMP-4 inhibits follicle-stimulating hormone secretion in ewe pituitary. J Endocrinol.

[45] Tata B, El N, Mimouni H, Barbotin A, Malone SA, Pigny P, Dewailly D, Catteau-jonard S, Sundström-poromaa I, Piltonen TT, Bello FD, Medana C, Prevot V, Clasadonte J (2018). Elevated prenatal anti-Müllerian hormone reprograms the fetus and induces polycystic ovary syndrome in adulthood. Nat Med.

[46] Thakare R, Chhonker YS, Gautam N, Alamoudi JA, Alnouti Y (2016). Quantitative analysis of endogenous compounds. J Pharm Biomed Anal.

[47] Thomas A, Geyer H, Kamber M, Schänzer W, Thevis M (2008). Mass spectrometric determination of gonadotrophin-releasing hormone (GnRH) in human urine for doping control purposes by means of LC-ESI-MS/MS. J Mass Spectrom.

[48] Grebe SKG, Singh RJ (2016). Clinical peptide and protein quantification by mass spectrometry (MS). TrAC - Trends Anal Chem.

[49] Thomas A, Schänzer W, Thevis M (2014). Determination of human insulin and its analogues in human blood using liquid chromatography coupled to ion mobility mass spectrometry (LC-IM-MS). Drug Test Anal.

[50] Thomas A, Krombholz S, Wolf C, Thevis M (2021). Determination of ghrelin and desacyl ghrelin in human plasma and urine by means of LC–MS/MS for doping controls. Drug Test Anal.

[51] Zhao Y, Gu H, Postelnek J, DeMichele M, Yuan L, Zhang YJ, Zeng J (2020). Fit-for-purpose protein biomarker assay validation strategies using hybrid immunocapture-liquid chromatography-tandem-mass spectrometry platform: quantitative analysis of total soluble cluster of differentiation 73. Anal Chim Acta.

[52] Dal Bello F, Lamberti C, Giribaldi M, Garino C, Locatelli M, Gastaldi D, Medana C, Cavallarin L, Arlorio M, Giuffrida MG (2021) Multi-target detection of egg-white and pig gelatin fining agents in Nebbiolo-based aged red wine by means of nanoHPLC-HRMS. Food Chem 345: . 10.1016/j.foodchem.2020.128822 10.1016/j.foodchem.2020.128822 33352406

[53] Owusu BY, Pflaum H, Garner R, Foulon N, Laha TJ, Hoofnagle AN (2021). Development and validation of a novel LC-MS/MS assay for C-peptide in human serum. J Mass Spectrom Adv Clin Lab.

[54] Kamphorst JJ, Van Der Heijden R, DeGroot J, Lafeber FPJG, Reijmers TH, Van El B, Tjaden UR, Van Der Greef J, Hankemeier T (2007). Profiling of endogenous peptides in human synovial fluid by nanoLC-MS: method validation and peptide identification. J Proteome Res.

[55] Jenkins R, Duggan JX, Aubry A, Zeng J, Lee JW, Cojocaru L, Dufield D, Garofolo F, Kaur S, Schultz GA, Xu K, Yang Z, Yu J, Zhang YJ, Faye V (2015) White paper recommendations for validation of LC-MS / MS bioanalytical methods for protein biotherapeutics. 17: . 10.1208/s12248-014-9685-5 10.1208/s12248-014-9685-5 PMC4287296 25392238

[56] Šesták J, Moravcová D, Kahle V (2015). Instrument platforms for nano liquid chromatography. J Chromatogr A.

[57] Olkowicz M, Rybakowska I, Chlopicki S, Smolenski RT (2015). Development and analytical comparison of microflow and nanoflow liquid chromatography/mass spectrometry procedures for quantification of cardiac troponin T in mouse hearts. Talanta.

[58] Vialaret J, Picas A, Delaby C, Bros P, Lehmann S, Hirtz C (2018). Nano-flow vs standard-flow: which is the more suitable LC/MS method for quantifying hepcidin-25 in human serum in routine clinical settings?. J Chromatogr B Anal Technol Biomed Life Sci.

[59] Caraty A, Locatelli A (1988). Effect of time after castration on secretion of LHRH and LH in the ram. J Reprod Infertil.

[60] Moore AM, Lohr DB, Coolen LM, Lehman MN (2021). Prenatal androgen exposure alters KNDy neurons and their afferent network in a model of polycystic ovarian syndrome. Endocrinol (United States).

